# Determining the Effects of Lifestyle Factors Including Smoking, Alcohol Consumption, Physical Activity, and Diet on the Risk and Progression of Dementia: A Systematic Review

**DOI:** 10.7759/cureus.108502

**Published:** 2026-05-08

**Authors:** Goodluck Oparaugo, Raashi Gaur

**Affiliations:** 1 Public Health, University of Derby, Derby, GBR

**Keywords:** alcohol consumption, cognitive impairment, dementia, diet, lifestyle factors, physical activity, smoking

## Abstract

Dementia and cognitive decline among older adults are increasingly considered as a significant health burden with a high level of personal, societal, and economic implications. Understanding the influence of lifestyle factors and potential lifestyle modifications on the risk and progression of dementia may aid in offering a non-pharmacological approach to reducing this public health challenge. This study explores the impact of key lifestyle factors - diet, physical activity, smoking, and alcohol consumption - on both the risk and progression of dementia. A systematic literature review was conducted using a predefined search strategy. Studies were identified through searches of the databases PubMed, Scopus, Web of Science, Embase, Medline, and CINAHL. Studies published between 2014 and 2024 were included to capture recent evidence. The quality of the selected studies was assessed using the Newcastle-Ottawa Scale (NOS) for cohort studies and the Joanna Briggs Institute (JBI) Critical Appraisal Checklist for cross-sectional studies. A total of 1,135 studies were initially identified; 12 met the inclusion criteria and were critically reviewed. The findings suggest that each lifestyle factor exerts an influence on the risk and progression of dementia, albeit to varying degrees. Further research is needed to deepen the understanding of these relationships and inform targeted preventive strategies.

## Introduction and background

Dementia is a major cause of individuals becoming dependent, especially among the elderly. This can pose significant economic, psychological, physical, and social effects on the affected individual and their relatives [1].

With increasing life expectancy worldwide, dementia and other dementia related conditions are increasingly becoming a major public health problem, with projections showing that the prevalence of dementia would nearly be quadrupled by 2040, by which time 1 in 45 Americans and 1 in 85 individuals worldwide would be affected [2]. Trends in dementia-related mortality further highlight the increasing public health significance. In the United States, while mortality attributed to heart disease, stroke, and human immunodeficiency virus (HIV) declined between 2000 and 2021, deaths related to Alzheimer's disease increased by more than 140% [3]. Modifying the effect of several lifestyle factors, including smoking, alcohol, physical activity, and diet, might prevent or delay dementia [4].

Existing evidence from different studies has shown that these factors may exert their effect through different mechanisms, including neurobiological pathways, by activating sensorineural networks in the prefrontal and hippocampal areas of the brain, hippocampal atrophy, reduction in brain volume, and interaction with genetic risk factor components, such as APOEe4 [5-8].

While several studies have addressed the effect of lifestyle factors on dementia, findings remain inconsistent, with some studies showing a positive association [6-8], while others report little or no impact [9,10]. With the burden of dementia increasing, and the potential for prevention through modifiable lifestyle factors, there is therefore a need for more research into specific predisposing and progression risk factors in order to develop a comprehensive and preventive strategy [2].

Therefore, this systematic review aims to evaluate the effects of lifestyle factors, including smoking, alcohol consumption, physical activity, and diet, on the risk and progression of dementia.

## Review

Methodology

Study Protocol and Research Question

This systematic review was conducted in accordance with the Preferred Reporting Items for Systematic Reviews and Meta-Analysis (PRISMA) guidelines. This study intends to answer the research question "How does lifestyle modification, including diet, exercise, smoking, and alcohol consumption, influence the risk and progression of dementia?" This was achieved by identifying and reviewing relevant literature and articles on this topic through a systematic review.

Search Terms and Databases

A combination of keywords and Medical Subject Headings (MeSH) terms was used to optimise the literature search strategy. The following search string was developed and adapted across different databases: (dementia OR cognitive decline OR neurodegenerative OR Alzheimer’s OR cognitive impairment) AND (smok*) AND (exercise OR physical activity) AND (alcohol*) AND (diet OR nutrition OR nutritional intake OR nutrient intake). Boolean operators (“AND” and “OR”) were applied to refine the search and ensure the retrieval of relevant studies. A systematic search of databases, including PubMed, Scopus, Web of Science, Embase, Medline, and CINAHL, was conducted via the University of Derby online library. Searches were restricted to studies published between 2014 and 2024 to ensure inclusion of recent evidence. All records retrieved after the search were imported into Zotero for duplicate removal and organisation. The literature search was conducted between 15th and 19th of June 2024 and updated between 24th of March and 3rd of April 2026 to ensure recent evidence was captured. Appendix 1 shows the full search strategy and specific adaptation for each database used.

Research Framework

This study employed the population, exposure, and outcome (PEO) framework to guide study selection and data synthesis. The population includes adults aged 40 years and above, irrespective of race or country of origin, as well as adults aged 65 years or older with a formal diagnosis of dementia, irrespective of race or country of origin. The exposure considered includes dietary patterns, nutrient intake and eating habits, physical activity and exercise levels, smoking status (current, former, or never smoked), and frequency of alcohol consumption. The primary outcomes involved dementia diagnosis and dementia progression.

Eligibility Criteria

Studies were included if they were observational, had investigated the impact of lifestyle factors (e.g., diet, physical activity, smoking, or alcohol consumption) on dementia risk or progression, and were published between 2014 and 2024. Those whose full texts were not available, not published in English, or not translatable into English were excluded.

Study Selection

**Figure 1:**
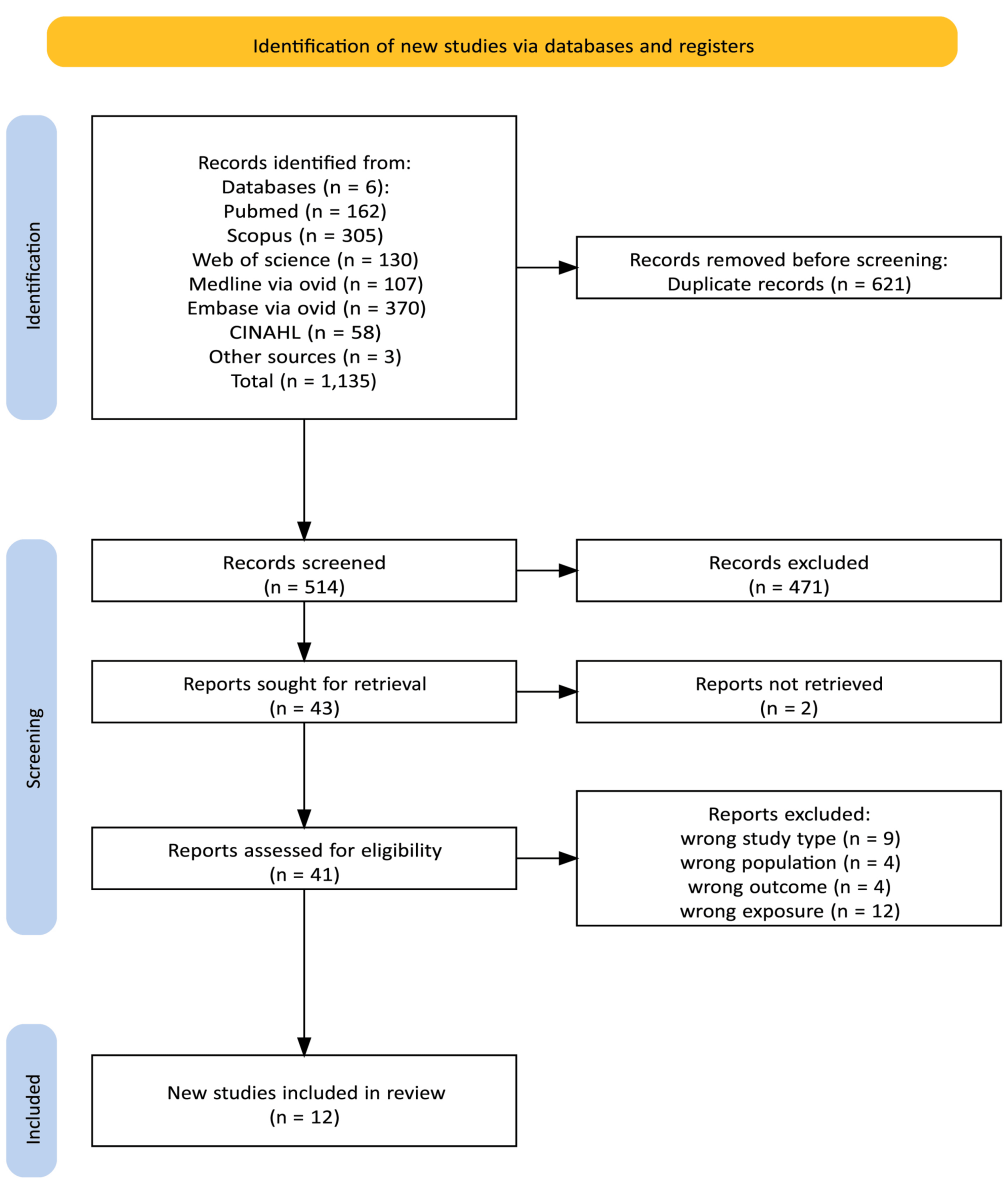
PRISMA flow chart showing the study selection process. PRISMA: Preferred Reporting Items for Systematic Reviews and Meta-Analyses

The study selection process was conducted in accordance with the PRISMA 2020 guidelines. Two reviewers, comprising the first reviewer (GO) and a senior researcher (RG), were involved in the independent data selection. Articles were screened based on the abstract, methods, and results. Studies were considered if they met the inclusion criteria; any disputes were discussed among the reviewers, and a consensus was reached. The studies were identified through database searches conducted through PubMed, Scopus, Web of Science, Embase, Medline, and CINAHL. In addition to database searches, a manual search of the reference lists of relevant studies and other sources was conducted to identify additional eligible studies. Three studies were identified, which met the inclusion criteria and were included. A total of 1,135 studies were identified: 1,132 from database searches and 3 from other sources. After removing duplicates, 514 records were screened. Of these, 471 were excluded for irrelevance or abstract screening, and 2 could not be retrieved. Forty‑one studies were assessed for eligibility: 9 were excluded due to wrong study type, 4 due to wrong population, 4 for not assessing dementia as the primary outcome, and 12 for using the wrong exposures. Ultimately, 12 studies met the eligibility criteria and were included in the study. The flow chart showing the study selection process is shown in Figure 1.

Data Extraction

Data were extracted from the selected studies using a standardised data extraction form developed by the review authors. The extracted information includes the author's name, year of publication, study design, sample size, population characteristics, exposure variables, outcome measures, key findings, and study limitations. The extracted information was then stored in Microsoft Excel for ease of analysis and organisation (Appendix 2). Data extraction was done initially by the first reviewer (GO), and the extracted data was then reviewed and verified by the senior researcher (RG), including the summary tables, to ensure accuracy and consistency. Due to the heterogeneity in the included studies, we did not perform a meta-analysis for this systematic review; instead, we used a structured narrative synthesis to analyse the data.

Protocol Registration

This review was not registered in PROSPERO or any other databases for systematic review; however, the study methodology was established in advance and conducted in accordance with PRISMA 2020 guidelines to maintain transparency and minimise bias.

Quality Assessment of the Included Studies

Appropriate appraisal tools were selected based on the study designs of the included articles. Cohort studies were assessed using the Newcastle-Ottawa Scale (NOS) [11], while cross-sectional studies were evaluated using the Joanna Briggs Institute (JBI) Critical Appraisal Checklist [12]. The risk of bias was initially assessed by the first reviewer and independently reviewed and verified by the second reviewer to ensure accuracy and consistency. Any disagreements that arose were resolved through discussion until a consensus was reached between the reviewers.

Using the JBL tool each of the included cross-sectional studies was evaluated in eight domains: D1 - inclusion criteria clearly defined, D2 - objective standard criteria employed in the measurement of condition, D3 - the exposure measured in a valid and reliable way, D4 - outcome measured in a valid and reliable way, D5 - confounding factors identified, D6 - strategies to deal with confounding factors rated, D7 - appropriate statistical analysis used, and D8 - study subject and setting described in details.

**Figure 2:**
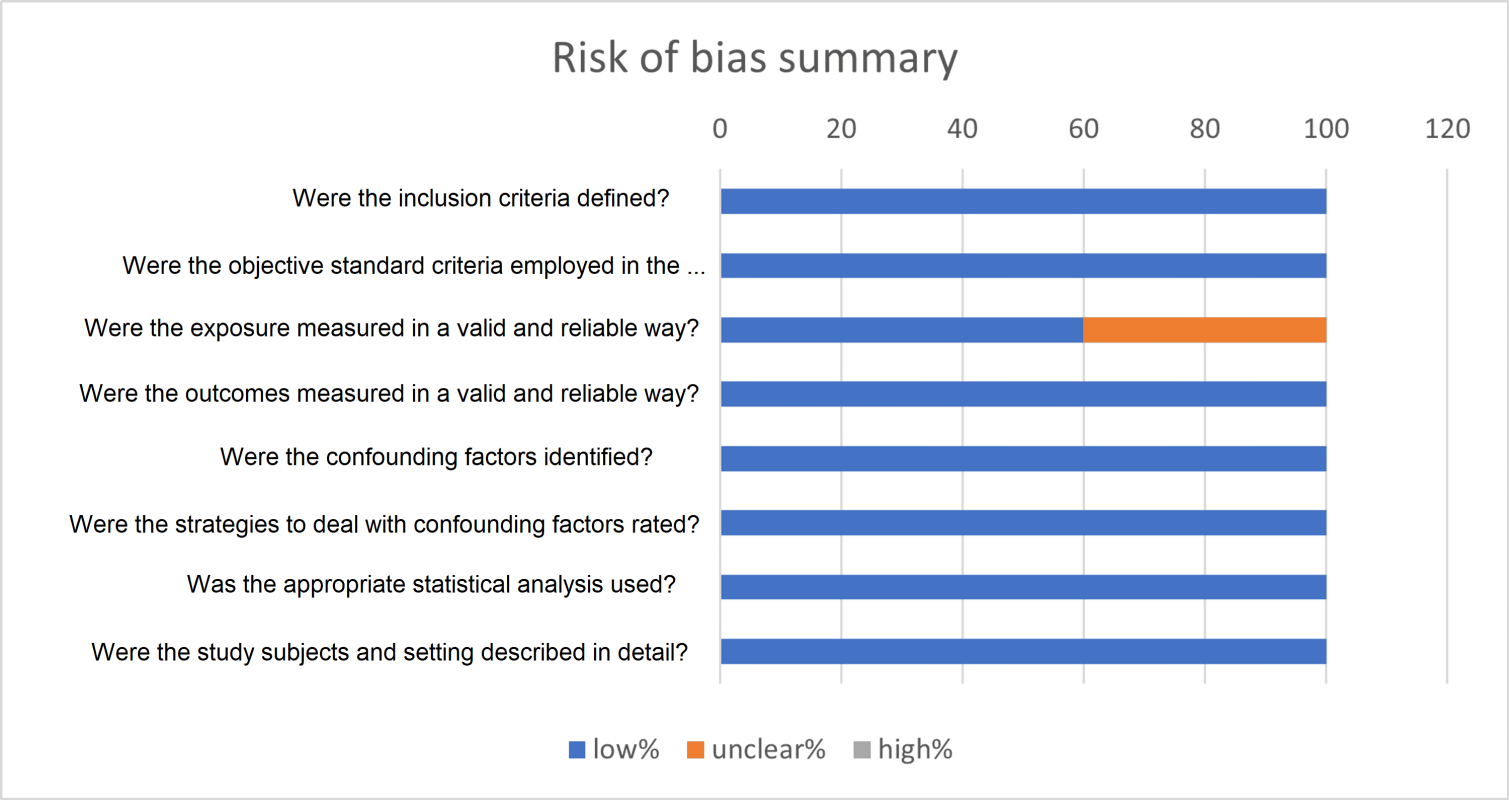
Summary of the overall risk of bias for cross-sectional studies. The figure illustrates the risk of bias assessment for the included cross-sectional studies using the Joanna Briggs Institute (JBI) tool. Each bar represents a specific quality criterion assessed and the proportion of studies that were rated "Yes", "No", or "Unclear" in the calculation of the overall risk for each domain.

The overall risk of bias for each study was appraised as low risk if all domains were clear and rated "Yes", intermediate risk if at least one domain was unclear, and high risk if at least one domain was rated "No". The selected papers were low to intermediate risk; none of the selected papers was high risk. The summary of risk of bias assessment for cross-sectional studies is illustrated in Figure 2 and Table 1.

**Table 1:** Risk of bias assessment for cross-sectional studies (JBI critical checklist tool). JBI: Joanna Briggs Institute The table demonstrates the quality assessment of included cross-sectional studies using the JBI tool, based on eight domains (D1-D8), each of which represents a quality criterion. Using the tool, "Yes", "No", or "Unclear" is assigned depending on how each study performs using the tool, with an overall risk assigned as low, high, or intermediate.

Study	D1	D2	D3	D4	D5	D6	D7	D8	Overall risk of bias
MacDonald-Wicks et al. [13] (2019)	Yes	Yes	Yes	Yes	Yes	Yes	Yes	Yes	Low
Rush et al. [14] (2017)	Yes	Yes	Yes	Yes	Yes	Yes	Yes	Yes	Low
Liu et al. [15] (2018)	Yes	Yes	Unclear	Yes	Yes	Yes	Yes	Yes	Intermediate
Jia et al. [16] (2024)	Yes	Yes	Unclear	Yes	Yes	Yes	Yes	Yes	Intermediate
Claire et al. [17] (2017)	Yes	Yes	Yes	Yes	Yes	Yes	Yes	Yes	Low

For the cohort studies, the NOS was used to assess methodological quality. A total of seven cohort studies were evaluated. Studies with ratings between six and nine stars were rated as good quality; those below six were rated as poor and were excluded from the study. Only studies rated as good quality were included in this study. One study achieved a total score of 7, four studies achieved a total score of 9, and two studies achieved a total score of 8. The summary for the overall risk of bias for cohort studies is shown in Table 2.

**Table 2:** Risk of bias assessment for cohort studies using the Newcastle-Ottawa Scale (NOS). The table summarises the risk-of-bias assessment for cohort studies using the Newcastle-Ottawa Scale. Studies were evaluated across different domains (selection, comparability, and outcome), scores represented by stars are assigned to each domain, and a total score is calculated, with higher scores indicating a low risk of bias.

Author (year)	Selection (max 4)	Comparability (max 2)	Outcome (max 3)	Total score	Quality rating
Rao et al. [18] (2021)	***	**	**	7/9	Good
Cheng et al. [19] (2023)	****	**	***	9/9	Good
Dhana et al. [20] (2022)	****	**	***	9/9	Good
Jia et al. [21] (2023)	****	**	***	9/9	Good
Kunustor et al. [22] (2020)	***	**	***	8/9	Good
Otuyana et al. [23] (2019)	****	**	***	9/9	Good
Deal et al. [24] (2019)	***	**	***	8/9	Good

Critical Appraisal of the Selected Studies

The critical appraisal process identified several methodological strengths across the included studies. Most studies clearly stated their research aims, study design, and population characteristics. Exposure variables, such as diet, physical activity, smoking, and alcohol consumption, were generally measured using validated questionnaires or standardised assessment tools. This includes food frequency questionnaire (FFQ), Willet FFQ, and 24-hour dietary recall for diet, Kuopio ischemic heart disease (KIHD) physical activity questionnaires, self-reported physical activity questionnaires, healthy life scores for physical activity, smoking status questionnaires for smoking, and alcohol use disorders identification test-consumption (AUDIT-C) and FFQ (for alcohol intake) for alcohol. Outcomes related to cognitive function and dementia diagnosis were assessed using recognised clinical or neuropsychological instruments, including the mini-mental state examination (MMSE), structured diagnostic criteria, and medical records.

Results

The study characteristics of the selected articles are presented in Table 3.

**Table 3:** Overview of selected Articles The table illustrates the important characteristics of included studies, showing the author, year of publication, research methods, and main findings of each included study. DSM-IV: Diagnostic and Statistical Manual of Mental Disorders, Fourth Edition

Article number	Author(s), year	Study design	Research question/aim/objectives	Sample method and/or sample size	Methodology	Main findings/conclusions
1	MacDonald-Wicks et al. (2019) [13]	Cross-sectional study	This study aimed to establish whether dietary long-chain fatty acids impact cognitive performance among older Australian adults.	Analysis of cross-sectional data from the hunter community study involving a random selection of participants aged 55-85 years. A total of 2,750 participants, comprising 1,198 men and 1,552 women, were analysed.	It involves a cross-sectional data analysis using the Hunter community study among older Australian adults who are aged 55-85 years, using a 145-item food frequency questionnaire (FFQ) to measure dietary intake, and mini-mental state examination (MMSE) and audio-recorded cognitive screening (ARCS) for measuring cognitive performance.	According to the authors, consumption of n-6 polyunsaturated fatty acids results in improved cognitive performance when assessed with the audio-recorded cognitive screening (ARCS) rather than the mini-mental state examination (MMSE), while n-9 monounsaturated fatty acids and saturated fatty acids (SFA) showed no significant relationship.
2	Rush et al. (2017) [14]	Cross-sectional study	The objective of this study is to assess the relationship between varying levels of dietary sodium intake and cognitive function among older adults.	A cross-sectional sample was drawn from the Rando-Bernardo study, which is a longitudinal study of cardiovascular disease risk factors. There were 925 participants recruited from the Rancho study, comprising 373 men and 552 women aged 50-96 years.	It involved a cross-sectional study design, with the setting in southern California communities. Exclusion criteria comprise those below 50 years of age and those with missing dietary data, cognitive function scores, or educational information. Data collection for dietary assessment of sodium intake was conducted using the Willett food frequency questionnaire (Willett FFQ), while cognitive assessment was conducted by trained interviewers using the verbal fluency test (VFT), mini-mental state examination (MMSE), and Trail Making Test Part B (Trail B). Covariate assessment was conducted using a standardised self-administered survey for demographic variables, clinical measurements, and blood biochemical analysis for plasma glucose and haemoglobin A1c.	The study could not find a relationship between higher intake of dietary sodium and reduced cognitive function, and lower sodium intake was however shown to have a poor outcome on cognition using the MMSE and Trial B; this effect was higher in the oldest elderly (age above 80 years).
3	Liu et al. (2018) [15]	Cross-sectional study	The study aimed to investigate the association among healthy lifestyle, gender, and cognitive function in older Chinese individuals.	Participants were recruited using stratified random sampling of age groups 65-74 years, 75-84 years, and older. A sample size of 1,831 community dwellers who are aged 65 years or above with no known dementia or other psychiatric disorders.	The researchers used a cross-sectional analysis in this study. The setting comprises Cantonese-speaking Chinese tenants, and the data collection method involved the use of the Cantonese Chinese Montreal cognitive assessment (CC-Mo CA) for assessing individual cognitive function. Lifestyle factors were assessed in 6 domains: dietary habits, smoking, alcohol consumption, exercise, and cognitive stimulating activity (using questions from the healthy ageing quiz).	The result of this study shows that being healthy in different lifestyle domains, also known as a comprehensive healthy lifestyle, is associated with better cognitive function in older individuals. This effect was more in women than in men. Physical activity and exercise showed a stronger association with cognitive function among older people compared with other domains. The relationship between healthy lifestyle factors also differed by gender.
4	Jia et al. (2024) [16]	Cross-sectional study	The study aimed to establish the relationship between dietary vitamin B1 intake and cognition in older adults.	Data from the National Health and Nutrition Examination Survey (NHANES) 2011-2014 were used. The sampling method for the NHANES involved a stratified multistage probability cluster sampling, which ensured appropriate representation of the US population. The final sample size comprises 2,422 participants who were aged 60 years and older.	A cross-sectional observational study design was used. Data collection used a 24-hour dietary recall questionnaire to assess vitamin B1 intake. This questionnaire was used to collect data on the type and amount of food consumed in the last 24 hours. Each participant was eligible for 2 dietary recall interviews: a face-to-face and a telephone interview, 3-10 days apart. The daily vitamin B1 intake was then measured by averaging result from the 2-24 hour recalls and grouping of participants into quartiles using these data. For the assessment of cognitive function, three different tools were used to assess cognition in different domains.	A relationship between vitamin B1 intake and improved cognitive function was established in this study.
5	Clare et al. (2017) [17]	Cross-sectional study	The research aimed to assess the mediating effect of cognitive reserve on the association between lifestyle factors and cognitive function.	Using a stratified random sampling method into 2 groups of 65-74 years and 75 and older, 2,315 study participants who are aged 65 years and over were analysed using data from Cognitive Function and Ageing Wales (CFAS-Wales). The sample size consisted of 3,315 participants from a baseline sample of 3,593 individuals. These participants were followed up for up to 2 years.	The researchers used a cross-sectional design in this study. The setting involved areas in Gwyedd, Ynys Mon, and Neath Port Talbot in Wales. Participants were included in the study if they were aged 65 years or older and were considered cognitively healthy according to the MMSE. Exclusion criteria comprised of individuals less than 65 years, those with cognitive impairment, dementia, or depression. Data collection was conducted through face-to-face interviews at the homes of individual participants between 2011 and 2013. Cognitive function was measured using the Cambridge Cognition Examination (CAMCOG), and cognitive reserve was measured using the level of education and occupation complexity. Lifestyle factors were measured using self-reported information on alcohol consumption, exercise level, smoking, diet, cognitive and sexual activities, and covariates such as age, gender, and chronic medical conditions were also put into consideration.	The findings from this study supported the already existing hypothesis that cognitive reserve plays a mediating role between the effect of lifestyle factors on cognition. Physical activity, light-to-moderate alcohol consumption, and a healthy diet showed a positive relationship with cognition. The research however could not establish a relationship between cognitive function and smoking status.
6	Rao et al. (2021) [18]	Cohort study	The research authors aimed to investigate the relationship between risky alcohol consumption and cognitive decline.	The study sample was drawn from the PROTECT study, comprising adults aged 50 or older. Participants were recruited online through national media and existing older adult cohorts. Each study participant provided consent via an online consent form; the initial sample size was 15,582 participants, and the follow-up sample size after 2 years was 5,316.	It involves an online cohort study comprising only UK residents who have access to the internet. Data collection was conducted using baseline questionnaires for demographic and medical history data. Alcohol use identification test-consumption (AUDIT-C) for scoring and measuring risky drinking, and cognitive function through the use of paired association learning test (PALT), self-ordered search test (SOS), digital span test (DS), and verbal reasoning test (VR), and, finally, informant questionnaires on cognitive decline and self-rated scale were used for measuring functional impairment.	The study concluded that an association between risky drinking and cognitive impairment in domains of grammatical reasoning, visual episodic memory, spatial working memory, and verbal memory may be small but still significant.
7	Cheng et al. (2023) [19]	Cohort study	This study aimed to assess the effect of a combination of healthy lifestyle factors on cognitive function in older adults.	The researchers utilised data from the Chinese Longitudinal Health Longevity Survey (CLHLS) to establish a panel dataset. This was used to investigate the relationship between healthy lifestyle factors and cognitive function among individuals in China. Through four waves of data collection. A total of 8,423 participants, selected by random sampling, aged 60-100 years, were included, and a total of 24,260 observations were made.	It involved a longitudinal panel data analysis, and data were collected and surveyed using internationally standardised questionnaires administered by highly trained health personnel. Independent variables in the study, comprising lifestyle modification components of smoking, alcohol consumption, diet, and physical activity, were assessed using the healthy lifestyle score, while the dependent variable, cognition, was measured with the MMSE scale.	The results suggest that the effect of a healthy lifestyle on cognition is more pronounced among women compared to men. Also, older individuals who possess higher lifestyle scores may show less cognitive decline. No association between smoking and cognitive function was observed; no effect of alcohol on cognition was observed; and exercise was found to have a positive association with cognitive performance. Overall, the study showed that, as age increases, a healthy lifestyle score may help in improving cognitive performance.
8	Dhana et al. (2022) [20]	Cohort study	The objective of this research was to establish the effect of a healthy lifestyle on dementia among two groups: those with Alzheimer’s dementia and others without dementia.	The study utilised a prospective cohort approach using participants from the Chicago Health and Ageing Project (CHAP) in the United States. A sample size of 2,449 individuals who are 65 years or older was drawn.	A population-based cohort design was used to investigate the impact of lifestyle factors on life expectancy among individuals with and without dementia. Data collection was done through an in-home-based assessment using structured self-administered or interview-based questionnaires and collection of bio specimens. Modifiable lifestyle factors were assessed using a healthy lifestyle score developed for the study across 5 key domains: diet, physical activity, smoking, alcohol consumption, and cognitive activities. And each domain was assessed as follows: diet was assessed using the MIND score (Mediterranean-DASH diet intervention for neurodegenerative delay), for cognitive activities, reading and participating in games, and time used for moderate exercise per week for physical activity. Alcohol consumption through FFQ questionnaires and smoking using smoker, current smoker, and former smoker. Within each category, 1 is assigned for good, healthy behavior, and 0 otherwise, with a score range of 0-5.	Individuals with a healthy lifestyle who achieved a high lifestyle factor score of 4-5 showed lower Alzheimer’s dementia risk compared with individuals with a low lifestyle score. However, there was an inverse relationship observed between a high lifestyle score and mortality risk among women with dementia, with no significant association noted in men. Overall, the study suggests that, in individuals without Alzheimer’s dementia. A healthy lifestyle was associated with lower Alzheimer’s risk, while in those with Alzheimer’s disease, healthy lifestyle factors were not associated with increased life expectancy.
9	Jia et al. (2023) [21]	Cohort study	The study objective was to identify the relationship between a healthy lifestyle and memory decline among older people.	A sample size of 29,072 was included with a follow-up time of 10 years using a multi-stage sampling method.	A population-based prospective cohort design was used across North, South, and West China. Participants included were aged 60 years or older, while people with dementia and mild cognitive impairment were excluded. Data were collected using group categorisation of 6 modifiable lifestyle factors into a favourable group (i.e., those with 4 or more healthy lifestyle factors), an average group (i.e., those with 2-3 lifestyle factors), and an unfavourable group (i.e., those with 0-1 factors). The lifestyle factors include healthy diet adherence, up to 150 minutes of moderate intensive exercise per week, active social contact, active cognitive association, never or former smoker, and never drank alcohol. Outcome was measured using the MMSE and an Auditory Verbal Learning Test (AVLT).	The study found that, in people who are genetically predisposed to dementia, that is, those with the APO-e genotype at baseline, a favourable lifestyle is associated with a reduction in the rate of cognitive decline compared with those with an unfavourable and average lifestyle. The findings were also the same in older adults with normal cognition who are not genetically predisposed.
10	Kunutsor et al. (2020) [22]	Cohort study	The study aimed to investigate the relationship between physical activity and long-term risk of developing dementia and Alzheimer’s disease.	The sample was drawn from the Kuopio ischaemic heart disease (KIHD) study, which is a population-based prospective study with a sample size of 2,394 comprising healthy men aged 42-61 years with adequate cognitive function who were randomly selected.	A population-based prospective cohort study was used with data from the KIHD study. The follow-up period was 24.9 years, with the setting in eastern Finland. Participants excluded before the final sample included those who died, had serious illnesses, migrated individuals, and declined informed consent. Data collection was through the use of the KIHD - for leisure time questionnaire (LTPA) with frequency, exercise time, and intensity over the past year. Occupational physical activity among those who are working was assessed using a self-reported questionnaire assessing the duration of sitting, standing, walking, and climbing. Data for the outcome Alzheimer’s and dementia were assessed and collected from the national registry, which covers all hospital admissions in Finland. Cognitive testing was done using the MMSE and geriatric mental state test.	There was no association observed between physical activity and risk of dementia. This result was also consistent with the risk of Alzheimer’s.
11	Otuyama et al. (2019) [23]	Cohort study	The research study aimed to investigate the association between tobacco smoking and risk of dementia in seven lower- and middle-income countries (LMICs).	The sample comprised 11,143 individuals aged 65 years or older without dementia.	This involved a secondary analysis of the 10/66 population-based cohort design. Follow-up lasted 3.8 years across 7 LMICs, including China, Cuba, the Dominican Republic, Mexico, Peru, Puerto Rico, and Venezuela. Dementia diagnosis was through DSM-IV and the 10/66 diagnosis protocol for LMICs through clinical interview, informant interview, and physical examination. Exposure to tobacco smoking was measured using smoking status and pack years smoked. Covariates such as sociodemographic status, physical impairment, and hazardous alcohol consumption were considered.	The findings from the results showed that a significant relationship exists between tobacco smoking and dementia, including Alzheimer’s disease.
12	Deal et al. (2019) [24]	Cohort study	This research objective was to establish the relationship between cigarette smoking, the timing of cessation of cigarette smoking, and dementia risk.	A total sample of 13,002 individuals aged 52-75 years was obtained.	A population-based cohort study of an ongoing atherosclerotic risk in communities (ARC) study in US communities.	There was an association between current cigarette smoking, recent cessation, and increased risk of all-cause dementia over 12 years. No relationship was found among individuals who quit for 9 years or more.

Study Design

Two major study designs were used in the included studies: cohort and cross-sectional studies. Rao et al., Cheng et al., Dhana et al., Jia et al., Kunustor et al., Otuyama et al., and Deal et al. used a cohort design in their studies to evaluate the different effects of various or a combination of lifestyle factors on cognition [18-23].

Five studies from this work, McDonald-Wicks et al., Rush et al., Lui et al., Jia et al., and Clare et al. used a cross-sectional design to establish an association between lifestyle factors and cognitive function or dementia [13-17]. This approach enabled the assessment of the relationship between lifestyle factors and dementia, thereby addressing the research aim.

Sampling Method and Sample Size

McDonald-Wicks et al., Cheng et al., and Kunustor et al. employed a simple random sampling [13,19,22], while Jia et al., Lui et al., and Claire et al. employed a stratified random sampling [15,17,21], and Jia et al. used a multistage cluster sampling [16]. The simple random sampling method is less sophisticated and gives every member of the target population an equal opportunity of being selected, thereby reducing selection bias [13,19].

The use of stratified random sampling ensured that the studies by Lui et al., Jia et al., and Claire et al. classified study participants into different groups, thereby resulting in a more accurate or precise estimate of the population [15-17].

Across the included studies, appropriate sample sizes were selected to ensure adequate representation of the study population. McDonald-Wicks et al. analysed 2,750 participants (1,198 men and 1,552 women) [13], Rao et al. analysed an initial sample of 15,582 (with a follow-up sample after two years of 5,316) [18], Cheng et al. selected 8,423 participants through four waves of data collection [19], and Dhana et al. sampled 2,449 individuals, who were 65 years or older [15].

Overall, the studies showed a large-sample trend, which can adequately enhance the generalizability of the research findings.

Data Collection Method

Different data collection methods were utilised across the included studies to ensure that the collected data were robust and relevant to the research objectives.

McDonald-Wicks et al. used a 145-item semi-quantitative FFQ to measure the dietary intake of fatty acids [13]. Prior validation and pilot testing of this questionnaire were conducted using a four-day weighted food record in elderly individuals. Transcription was done using an electronic database. Rao et al. administered a baseline questionnaire to collect sociodemographic data, and lifestyle modification factors, e.g., alcohol consumption, were assessed using the AUDIT-C, which was validated against alcohol use disorder in the older population [18]. While smoking was assessed using lifestyle questionnaires, there was no prior pilot testing mentioned. In the study by Cheng et al., data on lifestyle modification components were collected using the healthy lifestyle score, which was administered using the Chinese version of the MMSE questionnaire, developed in accordance with the international MMSE questionnaire [19]. Dhana et al. collected data on lifestyle factors and medical history via in-home assessment using interviewer-administered questionnaires and bio-specimen collection [20]. This included the use of FFQs to measure diet and alcohol consumption, and interview surveys for physical activity, with no pilot testing reported. In the study by Jia et al., data were collected using a healthy behaviour questionnaire that assessed six modifiable risk factors [21]. This was transcribed by re-categorising the collected data into favourable, unfavourable, and average categories. Using assigned scores, Rush et al. used the 153-item Willett FFQ for dietary assessment, the MMSE, the verbal fluency test (VFT), and the Trail Making Test Part B (Trial B) [14]. Covariate assessment was conducted using a self-administered, standardised survey. Kunustor et al. assessed physical activity data through trained interviews using the KIHD-physical activity questionnaire, which was modified from the Minnesota-LTPA questionnaire [22]. Outcomes were assessed using MMSE. In Liu et al.'s study, data collection on lifestyle factors was conducted using questions adapted from the healthy ageing quiz [15]. Cognition was assessed using the Cantonese Chinese Montreal Cognitive Assessment (CC-MoCA), with scores ranging from 0 to 30. Pilot testing of this tool was conducted by validating it through a local study, which suggested a score of 19/20 for Alzheimer's and 22/23 for mild cognitive impairment. In Jia et al.'s study, data on sociodemographic and health-related lifestyle factors were collected through health interviews, and dietary intake of vitamin B1 was recorded using a 24-hour food recall questionnaire administered during a face-to-face telephone interview [16]. Otuyama et al. collected data through a population-based survey, including clinical interviews, informant-based interviews, and physical examination [23]. Dementia diagnosis was done using the Diagnostic and Statistical Manual of Mental Disorders, Fourth Edition (DSM-IV) criteria and the 10/66 diagnosis procedure, which was validated using the 10/66 dementia research group for LMIC. Claire et al. collected data from CFAS-Wales through interviews conducted in participants' homes [17]; these interviews were transcribed in English and Welsh. Cognitive function was measured using the Cambridge Cognitive Examination (CAMCOG). This tool was originally designed for cognitive function assessment in the older population. Deal et al. collected data via in-home assessment using self-administered or interviewer-administered questionnaires and bio-specimen collection [24]. Transcription was done by recording responses from questionnaires into standard formats for analysis.

Validity and Generalizability

In the study by McDonald-Wicks et al., the cohort's homogeneity was ensured through the Hunter Community Study, which used a sample of community-dwelling adults aged 55-85 years [13]. From Newcastle, New South Wales, Australia, possible confounders such as age, gender, and BMI were identified, and their links to cognitive function and diet were also investigated. In the study by Rao et al., measurement of variables and outcomes was conducted using well-validated tools: MMSE, ARC, and FFQ [18]. Cheng et al. stated that confounding factors such as education, marital status, and age were controlled [19]. In Claire et al.'s study, due to the use of a cross-sectional study design, the study was able to establish an association [17]; this, in turn, limited causal inference from the study. Liu et al. clearly stated their inclusion and exclusion criteria and how missing data were accounted for to avoid bias [15]; Dhana et al. used a long follow-up period of 19 years to allow for the long-term effects of physical activity on cognition to be ascertained [20].

In the study by Deal et al., study participants were mainly white or black adults [24]; their findings, therefore, can only be generalised to settings with the same ethnic profile. In the study by Jia et al., the use of 2,422 participants enhances its generalizability [21]; however, the authors recognised the presence of residual confounding, which may reduce generalizability. Otuyama et al. used data collected in seven LMICs across 10 catchment sites [23]; this will therefore enhance its generalizability to other regions, especially lower- and middle-income countries (LMIC) with similar characteristics.

Reliability

The use of standardised measuring instruments across the included studies enhances the reliability of the findings. Additionally, several instruments were pilot tested.

McDonald-Wicks et al. utilised the FFQ, MMSE, and ARC, which are standardised instruments [13]; Rao et al. used the standardised AUDIT-C for alcohol consumption and the paired-association learning test (PALT) for cognitive function [18]. In the study by Kunustor et al., a large sample size of 2,394 and a follow-up period of 24.9 years enhanced its reliability [22]. However, other forms of dementia were not put into consideration, with only Alzheimer's and all-cause dementia considered; also, only middle-aged men of Caucasian ethnic profile were considered, reducing its generalizability. In Rush et al.'s study, the outcomes were measured using the VFT, MMSE, and Trial B test [14]. These multiple measures, therefore, help maintain consistency and account for variability in the study. In Liu et al.'s study, self-reporting of events may be the cause of the observed small effect of lifestyle factors on cognition [15]. In Jia et al.'s study, self-reporting of lifestyle factors may introduce measurement error [21]; however, a large number of study participants (29,072) and a follow-up period of up to 10 years enhance reliability.

After in-depth analysis of the 12 chosen research articles, four main themes were identified for discussion: alcohol consumption, smoking, diet, and physical exercise.

Discussion

Smoking

Findings from the included studies on smoking and the risk and progression of dementia were inconsistent. Cheng et al. reported no significant association between smoking and cognitive function [19]; similarly, Dhana et al. reported an increased life expectancy between both former smokers and non-smokers [20]. A similar pattern was observed in the study by Jia et al. [21], while Clare et al. also failed to establish a significant relationship between smoking and dementia [17]. In contrast, other studies reported an association with long-term smoking cessation compared to individuals who do not smoke or after long-term smoking cessation [23,24].

Evidence from other literature further supports the role of smoking in dementia. Zhong et al. reported a reduced risk among never smokers and former smokers [25]. Their findings suggested that carriers of the APOEe4 allele may have amplified the predisposition among smokers. Additionally, different biological mechanisms, such as cortical thinning, reduced brain volume, impairment in cognitive domains, such as memory and cognitive function, promotion of tau protein phosphorylation and deposition by nicotine, oxidative stress, and vascular damage leading to hypercoagulability among smokers, have been reported [26-28]. Some studies have also reported no association [29]. Variations in findings may be due to differences in exposure measurement and categorisation, which can lead to misclassification bias and to population differences, such as APOEe4 allele status. Moreover, smokers who die before developing dementia may underestimate the true association in the elderly population.

Alcohol

The relationship between alcohol consumption and the risk and progression of dementia remains complex. Evidence from the included studies suggests that alcohol can exert both protective and detrimental effects on cognition. Several included studies reported a strong association [17,18,20], while some suggest that the effect of alcohol on cognition is modified by genetic and individual life patterns, with individuals carrying the Apoe4 allele experiencing a more rapid memory decline [21]. Differences in study populations may reduce the generalizability of these findings, with studies conducted in Chinese populations [15,21] highlighting a stronger relationship with genetic and gender-related influences compared to studies from the United States and the United Kingdom [18,20]. Population differences, such as cultural drinking habits, genetic predispositions, and baseline health status, can affect comparability among studies. Additionally, variation in how alcohol consumption was measured, such as frequency, quantity, and categorisation into moderate, heavy, or light drinking, may be a contributing factor to observed differences.

Several studies have also reported the association between alcohol consumption and cognition. Rehm et al. reported that heavy alcohol use is associated with structural brain changes [30]; others recorded that excessive alcohol consumption, quantified as either greater than three to four drinks per day or greater than fourteen units per week, significantly increases the risk of cognitive decline [31,32]. In contrast to these findings, some studies have reported a rather protective effect, especially from moderate consumption through neuroprotective mechanisms, especially in relation to vascular dementia [8].

Exercise

Findings on the effect of physical activity on the risk and progression of dementia were heterogeneous across the included studies. Cheng et al., using longitudinal data from the Chinese Longitudinal Health and Longevity Survey, reported that regular exercise is positively associated with cognitive function across age groups and genders [19]. This finding was supported by Dhana et al. and Jia et al. [20,21]. Similarly, Liu et al. established that engaging in at least 30 minutes of moderate-to-vigorous physical activity per day enhances cognitive function; these results were also consistent with findings from Clare et al. using data from the cognitive function and ageing study Wales (CFAS-Wales) with varying intensity of physical exercise classified as mild, moderate, and vigorous activities [17]. In contrast, Kunustor et al., in a long-term prospective cohort study, were unable to establish a significant association despite assessing both leisure-time and occupational physical activity [22]; this disparity in findings highlights the complex nature of the relationship between physical activity and cognitive outcomes.

The heterogeneity in findings across included studies can be explained by differences in exposure assessment; for example, Kunustor et al. included both occupational and leisure-time physical activity, which may reduce the effects typically associated with a structured exercise regimen [22]. Moreover, reliance on self-reported measures of physical exercise might introduce recall bias and misclassification. Population differences may influence observed associations, due to variations in cultural lifestyle patterns with studies conducted in different geographical and structural settings, such as China [19,21] and the Western population [17,20,22].

Several studies have also reported findings on exercise and cognition or on dementia. Evidence from Norton et al. estimated that a substantial proportion of Alzheimer's disease cases may be attributable to modifiable factors such as physical inactivity [33]. Forbes et al. demonstrated that structured exercise programmes in individuals with dementia improve both cognitive function and activities of daily living [34]. Farina et al. concluded that physical activity interventions may also have a beneficial effect on cognition in individuals with Alzheimer's disease [35].

Diet

Findings on the effect of diet were mixed across the included studies. McDonalds-Wicks et al. reported a positive association between n-6 polyunsaturated fatty acids and improved cognitive function, but conversely observed no significant relationship with other fatty acids, including n-9 monounsaturated fatty acids [13]. Some studies demonstrated that adherence to an overall healthy dietary pattern is associated with favourable cognitive outcomes [19-21]; however, not all findings were consistent. Rush et al. could not establish a clear relationship between higher sodium intake and reduced cognitive function [14]; instead, their findings suggested that a lower sodium intake was associated with poorer cognitive outcomes among the elderly. Similarly, Liu et al. reported that individuals who have maintained a comprehensive, well-balanced diet as a measure of healthy lifestyle factors showed better cognitive outcomes [15]. Additionally, Jia et al., using NHANES data, reported that higher dietary vitamin B1 intake was associated with improved cognitive function among the aged [16].

The observed discrepancies can be attributed to differences in dietary assessment across studies. Some studies focused on individual nutrients, such as vitamin B1, sodium, or specific fatty acids [13,14,16], while others assessed overall dietary patterns [15,19,20]. These differences limit comparability, as single-nutrient approaches may not capture the combined effects of a whole diet, which better represents dietary patterns.

Other literature also supports the role of nutrition in cognitive performance. Ellouze et al. reported that dietary patterns, such as the dietary approach to stop hypertension (DASH) and Mediterranean diets, may reduce the dementia risk through mechanisms that modulate oxidative stress, inflammation, and insulin resistance, which are key biological mechanisms for neurodegeneration [36]. Similarly, Zhu et al. demonstrated that omega-3 fatty acids have a protective effect against cognitive impairment [37]. Stefaniak et al. further reported that diets rich in antioxidants, B vitamins, and polyunsaturated fatty acids, such as the Mediterranean and MIND diets, are associated with reduced dementia risk [38]. In addition, Mielech et al. highlighted that adequate intake of some antioxidant vitamins (A, B, C, D, and E) may slow dementia progression, while deficiencies may be associated with increased risk [39].

Limitations of the Study

The conclusions that were made in this study were drawn from the 12 selected articles used in this research study. It is possible that additional relevant studies examining the relationship between lifestyle factors and dementia risk or progression were not included in this review.

Some of the studies included in this review were based on homogeneous samples. The conclusions drawn from these papers' results may not be very accurate, as their findings may not be generalizable to other settings and populations.

Overall, across the selected studies, self-reporting of lifestyle factors was predominant; this may have resulted in recall bias and overestimation of the effects of the investigated lifestyle factors. Additionally, all causes of dementia and Alzheimer's were predominantly mentioned, with other types, including vascular and mixed dementia, marginally mentioned.

This review protocol was not prospectively registered, which may limit transparency and increase the risk of duplication. Several additional factors that may influence dementia risk and progression, such as depression and social support, were not considered in this review, and the effect of the only four factors considered might be overestimated.

Practice Recommendation

The first practice recommendation in this area will be to increase health promotion activity on the effects of lifestyle factors, such as smoking, drinking alcohol, physical activity, and adequate nutrition, on dementia. This can be achieved through a more systematic and in-depth needs assessment on the impact of dementia and the valuable effect of a favourable lifestyle. Links to policies such as the WHO action plan on physical activity 2018-2030, the WHO SAFER technical package on alcohol [40], the WHO framework on tobacco control, and the WHO best buy policy for reducing unhealthy diet [40]. Approaches and strategies here will include health education and community mobilisation strategies.

Regulation of smoking and alcohol through the promotion of policies such as the WHO Framework Convention on Tobacco Control and the WHO Technical Package on Alcohol will ensure that the detrimental effects of alcohol and smoking are mitigated [40].

The government should also invest more resources in community mobilisation strategies through their involvement and participation, funding campaigns and awareness, and conducting systematic evaluations to assess the impact of these measures.

Research Recommendation

This study made conclusions using findings from 12 selected papers, with two study designs, cross-sectional and cohort secondary studies, predominating. Future studies should focus more on primary research in this area and expand the scope to include a larger number of studies to draw clearer associations and effects.

More research should also be conducted in this field to plug the knowledge gap, especially on the effect of smoking on cognitive function.

Adequate research on this topic in LMIC is lacking; therefore, researchers should shine a brighter light on the effects of lifestyle factors on cognition and dementia in these locations to help mitigate the lifetime impact of these causes on individuals in LMIC.

## Conclusions

This systematic review highlights the significant role of lifestyle factors in influencing the risk and progression of dementia. The findings suggest that healthier lifestyle factors, such as exercise, improved diet, and moderate alcohol intake, are associated with a favourable cognitive outcome. However, observed inconsistencies across studies highlight the influence of population differences, genetic predispositions, methodological variations, and exposure assessment. Further research into these areas may help ease the need for pharmacological therapies and create alternative ideas for mitigating the risk and progression of dementia.

## Appendices

**Table 4:** Appendix 1: Search strategy and database used.

Database	Search strategy
Pubmed	(Dementia OR cognitive decline OR neurodegenerative OR Alzheimer’s OR cognitive impairment) AND (smok*) AND (exercise OR physical activity) AND (alcohol*) AND (diet OR nutrition OR nutritional intake OR nutrient intake)
Scopus	TITLE-ABS-KEY(Dementia OR “cognitive decline” OR neurodegenerative OR Alzheimer’s OR “cognitive impairment”) AND TITLE-ABS-KEY (smok*) AND TITLE-ABS-KEY (exercise OR physical activity) AND TITLE-ABS-KEY (alcohol*) AND TITLE-ABS-KEY (diet OR nutrition OR “nutritional intake” OR “nutrient intake”)
Web of science	TS=(Dementia OR cognitive decline OR neurodegenerative OR Alzheimer’s OR “cognitive impairment”) AND TS=(smok*) AND TS=(exercise OR “physical activity”) AND TS=(alcohol*) AND TS=(diet OR nutrition OR “nutritional intake” OR “nutrient intake”)
Medline	(Dementia OR "cognitive decline" OR neurodegenerative OR Alzheimer’s OR "cognitive impairment") AND smok* AND (exercise OR "physical activity") AND alcohol* AND (diet OR nutrition OR "nutritional intake" OR "nutrient intake")
Embase	(Dementia OR "cognitive decline" OR neurodegenerative OR Alzheimer’s OR "cognitive impairment") AND smok* AND (exercise OR "physical activity") AND alcohol* AND (diet OR nutrition OR "nutritional intake" OR "nutrient intake")
CINAHL	(Dementia OR "cognitive decline" OR neurodegenerative OR Alzheimer’s OR "cognitive impairment") AND smok* AND (exercise OR "physical activity") AND alcohol* AND (diet OR nutrition OR "nutritional intake" OR "nutrient intake")

**Table 5:** Appendix 2: Data extracted from the articles.

Authors name	Year of publication	Study design	Sample size	Population characteristics	Exposure variables	Outcome measures	Finding/Association	Study limitations	Journal	Country	Study aims
MacDonalds-Wicks et al. [13]	2019	Cross-sectional study	2750	55-85 years	Dietary intake-FFQ	MMSE, Audio-recorded cognitive assessment (ARCS)	mixed association	Potential for measurement error due to self-reported FFQ	Nutrients	Australia	This study aimed to establish whether dietary long-chain fatty acids impact cognitive performance
Rush et al. [14]	2017	Cross-sectional study	925	50-90 years	Dietary sodium-Willet FFQ	Verbal fluency test (VFT) MMSE Trial B	mixed association	A homogeneous population limits generalizability	Journal of Nutrition, Health and Ageing	USA	The objective of this study is to assess the relationship between varying levels of dietary sodium intake and cognitive function
Liu et al. [15]	2018	Cross-sectional study	1831	65-84 years	4 domains: alcohol, exercise, smoking, and dietary habits	CC-Mo-CA: Chinese, Montreal cognitive assessment	Association exists	Reliance on dichotomous self-report	Ageing and Mental Health	China	The study aimed to investigate the association among healthy lifestyle, gender, and cognitive function
Jia et al. [16]	2024	Cross-sectional study	2422	65-74 years	Dietary B1 intake: 24-hour recall		Association exists	Measurement and recall errors due to self-reported 24-hour dietary reviews	Journal of Translational Medicine	USA	The study aimed to establish the relationship between dietary vitamin B1 intake and cognition
Clare et al. [17]	2017	Cross-sectional study	3315	65 years and over	Alcohol, exercise, smoking, and dietary habits using face-to-face interview	Cambridge Cognition Examination (CAMCOG)	Association exists	Potential effect of reverse causality	PLOS Medicine	Wales	To determine the association between lifestyle factors and cognitive function
Rao et al. [18]	2021	Cohort study	15,582	50 years or over	Alcohol use identification-consumption (AUDIT-C)	Paired association learning test PALT, self-ordered test SOS, digital span test DS, verbal reasoning test VR.	Association exists	Possibility of low sensitivity and type 1 statistical error. Possibility of confounders due to noncoding of individual physical disorders and medication, no distinction between current and former smokers	Ageing and Mental Health	UK	Relationship between risky alcohol consumption and cognitive decline
Cheng et al. [19]	2023	Cohort study	8423	60-100 years	Lifestyle scores for alcohol, exercise, smoking, and dietary habits)	MMSE	Mixed association	Self-reporting questionnaires can cause non-response bias and recall bias, possibility of selection bias	Archives of Gastroenterology and Geriatrics	China	To assess the effect of a combination of healthy lifestyle factors on cognitive function
Dhana et al. [20]	2022	Cohort study	2449	65 years or older	Diet - MIND physical activity - moderate exercise per week. Alcohol - FFQ Smoker- current, former	Reading and participation in games	Mixed association	Assessment of lifestyle factors based on self-report could be prone to measurement error. Adherence to lifestyle factors was determined at baseline and not updated during follow-up.	British Medical Journal	USA	To establish the effect of a healthy lifestyle on dementia
Jia et al. [21]	2023	Cohort study	29,072	60 years or older	Alcohol, exercise, smoking, and dietary habits, active social contact, and active cognitive association	MMSE, AVLT: Auditory Verbal Learning Test	APO-e predisposition	Self-reporting lifestyle factors are prone to measurement errors. Exclusion of participants due to missing data or not returning for follow-up might lead to selection bias	British Medical Journal	China	To identify the relationship between a healthy lifestyle and memory decline
Kunustor et al. [22]	2020	Cohort study	2394	42-61 years	KIHD for leisure time questionnaire (LTPA)	MMSE, Geriatric Mental State Test	No association	Self-reporting of physical activity exposures can lead to misclassification and reporting bias. Regression dilution bias due to physical activity assessment at a single point in time	European Journal of Clinical Investigation	Finland	Aimed to investigate the relationship between physical activity and long-term risk of developing dementia and Alzheimer’s disease.
Otuyama et al. [23]	2019	Cohort study	11,143	65 years or older	Smoking status; Packed years	DSM-IV 10/66 diagnostic protocol	Strong association	Short duration of follow-up. Use of questionnaires can lead to recall bias	Ageing and Mental Health	China, Cuba, República Dominicana, México, Perú, Venezuela, Puerto Rico	Aimed to investigate the association between tobacco smoking and the risk of dementia
Deal et al. [24]	2019	Cohort study	13,002	52-75 years	Lifestyle scores for alcohol, exercise, smoking, and diet	Clinical evaluation, standard diagnostic criteria	Association exists	Self-report of lifestyle factors introduces recall bias. Causality limitation of observational study. Unmeasured cofounders may still exist	Journal of the American Geriatrics Society	USA	Aimed to investigate the association between tobacco smoking and the risk of dementia

## References

[1] Perng CH, Chang YC, Tzang RF (2018). The treatment of cognitive dysfunction in dementia: a multiple treatments meta-analysis. Psychopharmacology (Berl).

[2] Gao S, Burney HN, Callahan CM, Purnell CE, Hendrie HC (2019). Incidence of dementia and Alzheimer disease over time: a meta-analysis. J Am Geriatr Soc.

[3] Alzheimer’s Association (2024). 2024 Alzheimer's disease facts and figures. Alzheimers Dement.

[4] Livingston G, Huntley J, Sommerlad A (2020). Dementia prevention, intervention, and care: 2020 report of the Lancet Commission. Lancet.

[5] Morris MC, Tangney CC (2014). Dietary fat composition and dementia risk. Neurobiol Aging.

[6] Paillard T (2015). Preventive effects of regular physical exercise against cognitive decline and the risk of dementia with age advancement. Sports Med Open.

[7] Topiwala A, Allan CL, Valkanova V (2017). Moderate alcohol consumption as risk factor for adverse brain outcomes and cognitive decline: longitudinal cohort study. BMJ.

[8] Wiegmann C, Mick I, Brandl EJ, Heinz A, Gutwinski S (2020). Alcohol and dementia - what is the link? A systematic review. Neuropsychiatr Dis Treat.

[9] Khan I, Petrou S, Khan K (2019). Does structured exercise improve cognitive impairment in people with mild to moderate dementia? A cost-effectiveness analysis from a confirmatory randomised controlled trial: the dementia and physical activity (DAPA) trial. Pharmacoecon Open.

[10] Akbaraly TN, Singh-Manoux A, Dugravot A, Brunner EJ, Kivimäki M, Sabia S (2019). Association of midlife diet with subsequent risk for dementia. JAMA.

[11] (2026). The Newcastle-Ottawa scale (NOS) for assessing the quality of nonrandomised studies in meta-analyses. https://ohri.ca/en/who-we-are/core-facilities-and-platforms/ottawa-methods-centre/newcastle-ottawa-scale.

[12] Barker TH, Hasanoff S, Aromataris E (2026). The revised JBI critical appraisal tool for the assessment of risk of bias for analytical cross-sectional studies. JBI Evid Synth.

[13] MacDonald-Wicks L, McEvoy M, Magennis E, Schofield PW, Patterson AJ, Zacharia K (2019). Dietary long-chain fatty acids and cognitive performance in older Australian adults. Nutrients.

[14] Rush TM, Kritz-Silverstein D, Laughlin GA, Fung TT, Barrett-Connor E, McEvoy LK (2017). Association between dietary sodium intake and cognitive function in older adults. J Nutr Health Aging.

[15] Liu T, Luo H, Tang JY, Wong GH (2020). Does lifestyle matter? Individual lifestyle factors and their additive effects associated with cognitive function in older men and women. Aging Ment Health.

[16] Jia W, Wang H, Li C, Shi J, Yong F, Jia H (2024). Association between dietary vitamin B1 intake and cognitive function among older adults: a cross-sectional study. J Transl Med.

[17] Clare L, Wu YT, Teale JC, MacLeod C, Matthews F, Brayne C, Woods B (2017). Potentially modifiable lifestyle factors, cognitive reserve, and cognitive function in later life: a cross-sectional study. PLoS Med.

[18] Rao R, Creese B, Aarsland D, Kalafatis C, Khan Z, Corbett A, Ballard C (2022). Risky drinking and cognitive impairment in community residents aged 50 and over. Aging Ment Health.

[19] Cheng T, Zhang B, Luo L, Guo J (2023). The influence of healthy lifestyle behaviors on cognitive function among older Chinese adults across age and gender: evidence from panel data. Arch Gerontol Geriatr.

[20] Dhana K, Franco OH, Ritz EM (2022). Healthy lifestyle and life expectancy with and without Alzheimer's dementia: population based cohort study. BMJ.

[21] Jia J, Zhao T, Liu Z (2023). Association between healthy lifestyle and memory decline in older adults: 10 year, population based, prospective cohort study. BMJ.

[22] Kunutsor SK, Laukkanen JA, Kauhanen J, Willeit P (2021). Physical activity may not be associated with long-term risk of dementia and Alzheimer's disease. Eur J Clin Invest.

[23] Otuyama LJ, Oliveira D, Locatelli D (2020). Tobacco smoking and risk for dementia: evidence from the 10/66 population-based longitudinal study. Aging Ment Health.

[24] Deal JA, Power MC, Palta P (2020). Relationship of cigarette smoking and time of quitting with incident dementia and cognitive decline. J Am Geriatr Soc.

[25] Zhong G, Wang Y, Zhang Y, Guo JJ, Zhao Y (2015). Smoking is associated with an increased risk of dementia: a meta-analysis of prospective cohort studies with investigation of potential effect modifiers. PLoS One.

[26] Durazzo TC, Mattsson N, Weiner MW (2014). Smoking and increased Alzheimer's disease risk: a review of potential mechanisms. Alzheimers Dement.

[27] Edwards Iii GA, Gamez N, Escobedo G Jr, Calderon O, Moreno-Gonzalez I (2019). Modifiable risk factors for Alzheimer’s disease. Front Aging Neurosci.

[28] Choi D, Choi S, Park SM (2018). Effect of smoking cessation on the risk of dementia: a longitudinal study. Ann Clin Transl Neurol.

[29] Abner EL, Nelson PT, Jicha GA, Cooper GE, Fardo DW, Schmitt FA, Kryscio RJ (2019). Tobacco smoking and dementia in a Kentucky cohort: a competing risk analysis. J Alzheimers Dis.

[30] Rehm J, Gmel GE Sr, Gmel G (2017). The relationship between different dimensions of alcohol use and the burden of disease - an update. Addiction.

[31] Neafsey EJ, Collins MA (2011). Moderate alcohol consumption and cognitive risk. Neuropsychiatr Dis Treat.

[32] Sabia S, Fayosse A, Dumurgier J (2018). Alcohol consumption and risk of dementia: 23 year follow-up of Whitehall II cohort study. BMJ.

[33] Norton S, Matthews FE, Barnes DE, Yaffe K, Brayne C (2014). Potential for primary prevention of Alzheimer’s disease: an analysis of population-based data. Lancet Neurol.

[34] Forbes D, Forbes SC, Blake CM, Thiessen EJ, Forbes S (2015). Exercise programs for people with dementia. Cochrane Database Syst Rev.

[35] Farina N, Rusted J, Tabet N (2014). The effect of exercise interventions on cognitive outcome in Alzheimer's disease: a systematic review. Int Psychogeriatr.

[36] Ellouze I, Sheffler J, Nagpal R, Arjmandi B (2023). Dietary patterns and Alzheimer’s disease: an updated review linking nutrition to neuroscience. Nutrients.

[37] Zhu RZ, Chen MQ, Zhang ZW, Wu TY, Zhao WH (2021). Dietary fatty acids and risk for Alzheimer's disease, dementia, and mild cognitive impairment: a prospective cohort meta-analysis. Nutrition.

[38] Stefaniak O, Dobrzyńska M, Drzymała-Czyż S, Przysławski J (2022). Diet in the prevention of Alzheimer’s disease: current knowledge and future research requirements. Nutrients.

[39] Mielech A, Puścion-Jakubik A, Markiewicz-Żukowska R, Socha K (2020). Vitamins in Alzheimer’s disease - review of the latest reports. Nutrients.

[40] (2026). The top 10 causes of death. https://www.who.int/news-room/fact-sheets/detail/the-top-10-causes-of-death.

